# Stroke: An electromyographic approach to the masseter and temporal muscles, orofacial soft tissue pressure, and occlusal force

**DOI:** 10.1371/journal.pone.0282362

**Published:** 2023-03-01

**Authors:** Robson Felipe Tosta Lopes, Marcelo Palinkas, Gabriel Pádua da Silva, Edson Donizetti Verri, Isabela Hallak Regalo, Camila Rosa Gonçalves, Jaime Eduardo Cecilio Hallak, Guilherme Gallo Costa Gomes, Simone Cecílio Hallak Regalo, Selma Siéssere

**Affiliations:** 1 Department of Basic and Oral Biology, Ribeirão Preto School of Dentistry, University of São Paulo, São Paulo, Brazil; 2 Faculty of Medicine of Ribeirão Preto, Department of Neuroscience and Behavioral Sciences, University of São Paulo and National Institute and Technology—Translational Medicine (INCT.TM), São Paulo, Brazil; Emory University, UNITED STATES

## Abstract

Stroke is a cerebrovascular disease that triggers changes in the central and peripheral nervous systems, and can compromise human body function. This cross-sectional observational study aimed to analyze the electromyographic (EMG) activity of the masseter and temporal muscles, orofacial soft tissue pressure, and strength of occlusal contacts in patients who had suffered a stroke. Twenty-four patients were divided into two groups: stroke (n = 12) and control (n = 12). The EMG of the masseter and temporal muscles was evaluated during mandibular rest, protrusion, right laterality, left laterality, and maximal voluntary contraction. The Iowa Oral Pressure Instrument (IOPI) was used to measure pressure from the tongue, lips, and buccinator muscles. A computerized system for occlusal analysis (T-Scan III) was used to measure the occlusal contact points of the right and left hemiarches (upper and lower) and the upper and lower first molars. Data were subjected to Student’s t-test (p < 0.05). The stroke group had lower normalized electromyographic activity, with a significant difference in the left temporal muscle during rest (p = 0.03) when compared to the control group. There was a significant difference between the groups in tongue pressure (p = 0.004) with a lower mean value in the stroke group. There was a significant difference between the groups in the evaluation of the occlusal contact points of the first permanent molars, with a lower mean percentage in the stroke group. The results indicate that stroke negatively affects functional performance of the stomatognathic system.

## Introduction

Stroke is defined as a focal or sometimes global neurological impairment, of sudden occurrence [1], constituting the second leading cause of mortality worldwide [2]. Deaths and disabilities from stroke occur with a higher incidence in low- and middle-income countries [3]. An upward trend in stroke mortality rates has been seen in middle-aged patients in the US, Brazil, China and European countries [4,5].

It is classified as ischemic when clot formation occurs, blocking blood flow to an area of the brain, which can occur in lacunar, thrombotic, and embolic forms. In the hemorrhagic type, there is a rupture of the blood vessel, causing hemorrhage in the brain structures, sudden deterioration of consciousness and neurological dysfunction, which may be intracerebral and subarachnoid [6–8].

Both types of strokes promote sequelae and complications in the human body, such as motor deficits, loss of balance, changes in strength of the upper and lower limbs, and spasticity. These factors can trigger motor and biomechanical disorders, contributing to the development of dysfunctions in the static and dynamic structures of the stomatognathic system [9]. According to the study by Gomes et al. [10], stroke compromises the dynamic functions of the stomatognathic system, especially when observing bite force and thickness of the masticatory muscles. Jian et al. [11] reported that the function of the masticatory muscles is affected after stroke and surface electromyography (EMG) was used to determine the functional patterns related to the neural activity of the deficient masticatory muscles.

Given the above and the possible implications of stroke on the stomatognathic system, this study aimed to analyze the EMG of the masseter and temporal muscles, pressure of orofacial soft tissues, and strength of occlusal contacts in patients who had suffered a stroke. The null hypothesis of this study was that stroke does not promote functional changes in the stomatognathic system.

## Material and methods

### Ethical approval

This cross-sectional observational study was approved by the Ethics Committee of the University of São Paulo at Ribeirão Preto Dental School (# 92222318.8.0000.5419). The informed consent form was signed by the patients.

### Study population

Patients were recruited from the Department of Basic and Oral Biology of the Faculty of Dentistry of Ribeirão Preto, University of São Paulo, the health care center of the physiotherapy clinic of UNIFAFIBE, the physiotherapy clinic of the Claretiano Centro Universitário, and the multidisciplinary block of the University of Ribeirão Preto, São Paulo, Brazil.

### Study design and patients

Initially, 45 patients diagnosed with stroke were selected based on a diagnosis confirmed by neurologists. Following the inclusion and exclusion criteria, a convenience sample was used, in which 12 patients (6 men and 6 women, aged 30‒80 years) were recruited, of which 9 were affected by the disease on the right side and 3 on the left side, from health care centers in communities in the cities of Ribeirão Preto, Bebedouro, Batatais, and region. The group without the disease, considered a control, was matched subject to subject by age, sex, and body mass index with the group with stroke (Table 1).

**Table 1:** Differences in characteristics (mean values ± standard deviation) between groups.

Groups	Age	Body Mass Index
SG	56.1 (±4.0)	28.6 (±1.0)
CG	54.0 (±3.9)	28.5 (±1.4)
p value	0.71	0.85

SG, stroke group; CG, control group. Significant difference, Student’s t-test (p < 0.05).

The inclusion criteria were as follows: > 5 years since the time of diagnosis; being in the clinical and drug treatment phase; natural dentition; and presence of first permanent molars. The exclusion criteria were as follows: presence of other functional and degenerative alterations, ulcerations, open wounds or skin hypersensitivity, cognitive alterations, and temporomandibular disorders.

To ensure that the convenience sample was representative, the sample calculation (pos hoc power analysis), was performed using G* Power 3.1.9.2 software (Franz Faul, Kiel University, Kiel, Germany). To perform this calculation, the values obtained at maximum tongue pressure (mean and standard deviation) for the groups with stroke and control were considered, which were respectively: 33.33 (± 17.61) and 55.66 (± 16 .47) considering the error of 5%. After the calculation, an effect size of 1.30 and a test power of 92% were obtained.

Only one trained researcher was responsible for the data collection. Personal protective equipment, such as procedure gloves, hats, masks, and disposable gowns, were used throughout the clinical examination due to the COVID-19 pandemic.

### Electromyographic activity analysis

To capture electromyographic signals from the masseter and temporal muscles, a Myosystem-Br1 P84 portable electromyograph (DataHominis Tec. Ltd, Brazil) was used. Differential simple active electrodes and a reference electrode on the wrist consisting of an oval stainless-steel plate, 45 mm long, 30 mm wide, and 1 mm thick, wrapped in plastic, were used.

Before placing the electrodes, the skin was cleaned with alcohol to eliminate grease and pollution residues. The electrodes were positioned by the same trained examiner. To ensure the correct location of the masticatory muscles, specific maneuvers of maximal voluntary contraction were performed, accompanied by digital palpation, according to the recommendations of the surface EMG for non-invasive assessment of muscles (SENIAM) project [12].

Electromyographic activity (microvolts) was evaluated during mandibular rest (4 s), protrusion (10 s), right laterality (10 s), left laterality (10 s), and dental clenching during maximum voluntary contraction (4 s). During the recording of electromyographic activities, the environment was kept calm and silent, with patients seated in a comfortable chair, upright posture, soles of the feet resting on the ground, and hands resting on the thighs. The head was positioned with the Frankfurt horizontal plane parallel to the ground [13].

### Orofacial soft tissue pressure analysis

The Iowa Oral Pressure Instrument model 2.3 (IOPI Medical, Redmont, WA, USA) was used to assess lingual pressure, the buccinator muscles (right and left), and the lips (upper and lower). This evaluation instrument consisted of a blue plastic bulb 3.5 cm in length, filled with air, and connected by a pressure transducer using an 11.5 cm plastic tube. The pressure values were measured in kilopascals (kPa) [14].

In the analysis of lingual pressure, the plastic bulb was inserted and positioned posterior to the palatal aspect of the anterior teeth, specifically in the region of the maxillary central incisors. Tongue pressure was exerted against the hard palate, asking the patient to raise the tongue until it touched the plastic bulb, pressing it with maximum pressure for an average time of three seconds.

To measure the pressure of the buccinator muscles, the patient was asked to press the bulb with the buccal mucosa against the vestibular region of the posterior teeth, for three seconds. To measure the lip pressure, the plastic bulb was positioned between the upper and lower lips, and the patient pressed it for three seconds. In all conditions, three collections were performed, and the highest value was used for the statistical analysis [15].

### Strength of occlusal contacts analysis

The T-scan III system (Tekscan Inc., Ann Arbor, MI, USA) was used to analyze the occlusal contact points of the right and left hemiarches (upper and lower) and the upper and lower first molars. During the occlusion analysis, there were no interferences due to the small thickness of the sensor on which the occlusal contacts were recorded, making it possible to determine the correlation of the occlusal surfaces according to the percentage of force.

Prior to occlusal analysis, the bite guide of the appliance was tested in the oral cavity. Soon after this procedure, the evaluator in charge measured the width of the central incisors (upper and lower incisors) using a digital caliper. The sensor was inserted between the upper and lower arches, aligning the position guide located on the support such that it was centered and fitted between the upper central incisor teeth.

The patients remained seated, with the upper limbs supported, spine erect, and head in a horizontal position, and were instructed by the evaluator to bite three times, pressing the sensor to reach 95 to 100% of the maximum force of the occlusal contacts. None of the patients reported any muscle or joint pain caused by clenching. The data obtained showed the maximum forces of occlusal contacts (%) of each patient [16].

### Statistical analysis

The raw electromyographic signal was used to derive values of electromyographic amplitudes, obtained by calculating the square root of the mean (RMS). RMS of dental clenching at maximum voluntary contraction was used for data normalization. Data were statistically analyzed using GraphPad Prism 5.0 program (GraphPad Software, Inc.). The results were obtained using descriptive analysis (mean and standard error) for each variable. After verifying the normal distribution of the sample (Shapiro-Wilk), Student’s t-test (p < 0.05) was used.

## Results

There was no difference between left and right side muscles EMG in the stroke group. Table 2 shows the normalized EMG results for the masseter and temporal muscles between the groups. The group with stroke had lower electromyographic activity, with the left temporal muscle showing a significant difference (p = 0.03) during the jaw-rest task when compared to the control group.

**Table 2:** Differences in mean values (± standard error) of normalized electromyographic data between groups.

Mandibular task Muscles	Groups		p value
	SG	CG	
	mean (±standard error)	mean (±standard error)	
Rest			
RM	0.24 (±0.07)	0.37 (±0.10)	0.30
LM	0.25 (±0.05)	0.36 (±0.12)	0.43
RT	0.34 (±0.09)	0.40 (±0.10)	0.68
LT	0.21 (±0.04)	0.41 (±0.08)	0.03
Protrusion			
RM	0.55 (±0.11)	0.51 (±0.11)	0.80
LM	0.49 (±0.08)	0.66 (±0.21)	0.46
RT	0.42 (±0.09)	0.33 (±0.07)	0.42
LT	0.30 (±0.08)	0.47 (±0.11)	0.24
Right Laterality			
RM	0.32 (±0.09)	0.45 (±0.12)	0.39
LM	0.53 (±0.13)	0.46 (±0.11)	0.71
RT	0.52 (±0.13)	0.37 (±0.05)	0.32
LT	0.24 (±0.05)	0.35 (±0.12)	0.27
Left Laterality			
RM	0.47 (±0.09)	0.51 (±0.07)	0.70
LM	0.54 (±0.14)	0.43 (±0.13)	0.59
RT	0.36 (±0.07)	0.32 (±0.05)	0.68
LT	0.38 (±0.07)	0.55 (±0.12)	0.24

SG, stroke group; CG, control group; RM, right masseter; LM, left masseter; RT, right temporalis; LT, left temporalis. Significant difference, Student’s t-test (p < 0.05).

The data on orofacial soft tissue pressures are shown in Table 3. There was a significant difference in tongue pressure (p = 0.004) with a lower mean in the stroke group.

**Table 3:** Differences in mean values (± standard error) of orofacial soft tissue pressure (kPa) between groups.

Variables	Groups		p value
	SG	CG	
	mean (standard error)	mean (standard error)	
Tongue	33.33 (±5.08)	55.66 (±4.75)	0.004
Right buccinator muscle	19.08 (±2.50)	23.00 (±1.12)	0.17
Left buccinator muscle	20.78 (±2.47)	23.83 (±0.80)	0.25
Lips	24.48 (±3.09)	26.75 (±2.95)	0.60

SG, stroke group; CG, control group. Significant difference, Student’s t-test (p < 0.05).

The comparative results between the right and left hemiarches (upper and lower) in the stroke and control groups are shown in Table 4. The stroke group showed significantly greater strength on the left side than on the right side (p = 0.03).

**Table 4:** Differences in mean values (± standard error) of occlusal contact force (%) on the right and left sides (upper and lower hemiarch) sides for both groups.

Upper and Lower Hemiarch	SG	CG
Right side	39.6 (±6.0)	52.9 (±3.6)
Left side	60.3 (±6.1)	47.3 (±3.6)
p value	0.03	0.31

SG, stroke group; CG, control group. Significant difference, Student’s t-test (p < 0.05).

Table 5 shows the data on the occlusal contact strength of the right hemiarch (upper and lower), left hemiarch (upper and lower), and first permanent molars (upper and lower) between the groups. There was a significant difference in the analysis of occlusal contact points for the first permanent molars, with a lower mean percentage for tooth 16 (p = 0.02), 26 (p = 0.001), 36 (p = 0.02) and 46 (p = 0.02) in the stroke group.

**Table 5:** Differences in mean values (± standard error) of occlusal contact force (%) of the dental hemiarch (right and left) and permanent first molars (upper and lower) between groups.

Occlusal Contact Force	Groups		p value
	SG	CG	
	mean (standard error)	mean (standard error)	
Hemiarch (right)	39.60 (±6.08)	52.69 (±3.67)	0.08
Hemiarch (left)	60.32 (±6.12)	47.31 (±3.67)	0.08
Upper molar (right)	9.23 (±3.43)	19.36 (±2.42)	0.02
Upper molar (left)	8.02 (±2.29)	19.63 (±2.01)	0.001
Lower molar (right)	11.23 (±2.62)	20.14 (±2.45)	0.02
Lower molar (left)	13.50 (±3.98)	24.75 (±2.13)	0.02

SG, stroke group; CG, control group. Significant difference, Student’s t-test (p < 0.05).

## Discussion

In this study, the stomatognathic system of patients who had suffered a stroke was analyzed to elucidate the possible deficits and functional effects of stroke on this complex system. For standardization, all patients included in the study sample were analyzed five years after the onset of the disease and were compared to a control group. The null hypothesis of this study was rejected because the stroke group showed functional changes in the stomatognathic system.

Data obtained at rest showed that patients who had suffered a stroke had lower normalized electromyographic activities for all analyzed muscles. Notably, this activity was significantly lower in the left temporal muscle than in the control group. These results agree with those of Jian et al. [17], who observed significantly lower electromyographic means for the right and left temporal muscles and the right masseter in patients with stroke than in young patients.

The results obtained for the stroke group differ from those of previous studies, which have shown that in healthy patients, during rest, the activity of the temporal muscles is greater than that of the masseter muscles [13]. It is believed that due to the force of gravity, the temporal muscles have a greater number of fibers recruited to maintain the postural activity of the mandible. It is possible that our results showed less significant differences between the groups because the samples of both the groups were matched patient to patient for age, sex and body mass index.

Clinically, greater activity of the masseter muscles and lesser activity of the temporal muscles are expected in protrusion [18]. Although the stroke group presented different values from the control group, these were not significant. In addition, the stroke group showed higher normalized electromyographic activity for the masseter muscles than for the temporal muscles, suggesting that despite the disease, the pattern of muscle function was similar to that of the control group.

On comparison between the groups regarding right laterality, no significant differences were found between the values of the normalized electromyographic means obtained in the different muscles, and the stroke group showed a pattern of muscle function similar to that of the control group. In this postural condition, greater activation of the masseter muscle contralateral to the movement is expected; that is, the left masseter muscle must be more active than the right masseter muscle. However, the opposite occurs for the temporal muscles, that is, the temporal muscle on the side of the movement must be more active than the contralateral muscle [19,20].

In left laterality, the normalized electromyographic means differed between the two groups; however, there was no significant difference. In this type of movement, the masseter muscle on the right side must be more active than that on the left, and the left temporal must be more active than the right temporalis [19,21], as shown in the functional performance of the control group. The same pattern of muscle activation was not observed in the stroke group, as the right masseter muscle had higher electromyographic means than the left masseter muscle.

The clinical changes in the muscle functioning pattern, both in mandibular rest and for left laterality, may be due to the stroke causing muscle abnormalities through a set of changes (denervation, disuse, remodeling, and muscle spasticity), which are responsible for the change in the phenotypic pattern and muscle atrophy [22].

In this study, comparison of maximal pressure of the lips, buccinator muscle, and tongue between the two groups showed lower tongue pressure in the stroke group. These results are in agreement with those of Moon et al [23]. and Galek et al. [24] who found lower lingual pressure in post-stroke patients.

Neurological diseases directly and indirectly affect the functions of the body, leading to deficits in all functional aspects. Speech, suction, and swallowing are performed with the direct participation of the tongue, lips, and buccinator muscles [25]. The lower pressure of the tongue prevents the control of the food bolus and increases the amount of oral residues, which increases the risk of aspiration [26]. In addition, changes in the tongue due to the occurrence of neurological diseases can promote dysphagia, sucking, and communication difficulties [25].

Previous studies have demonstrated a relationship between the size of the brain lesion and the brain structures involved, which may explain the decreased lingual pressure observed in our study. Lesions of the cerebral cortex that involve the precentral gyrus can compromise motor and sensory functions of the face, lips, and tongue, which may also affect pharyngeal peristalsis [27,28]. Thus, it is assumed that the patients in this study had their precentral gyrus area affected, compromising the performance of the tongue.

By analyzing the forces of the occlusal contact points, when comparing the same sides of the different groups, no significant difference was observed. However, when comparing the right and left sides, the stroke group showed occlusal imbalance with a higher percentage of occlusal contact force on the left side. These results are justified by the fact that impairment caused by plegia or paresis can compromise the face, causing deviation of the mandible due to spasticity and hypertonia, consequently resulting in asymmetry of the dental arches in patients with neurological diseases [28].

Further, when comparing the maxillary permanent molars (teeth 16 and 26) and mandibular permanent molars (teeth 36 and 46) between the groups, a much lower mean percentage of force was observed in the stroke group. Studies have shown that masticatory performance improves with an increase in occlusal force in the molar region and number of occlusal contact points, and with greater movement of the mandible in the vertical and horizontal directions [29]. Therefore, considering these findings and our study results, we can infer those patients in the stroke group had lower masticatory performance, as evidenced by the significantly lower percentage of molar occlusal force than those in the control group.

This study had some limitations. The sample size was small because of a specific inclusion criterion, which is the presence of first permanent molars, as these teeth are often not present in the oral cavity of older Brazilian patients. In addition, the data obtained in this study correlated with muscle spasticity; however, this was not measured during the study.

In conclusion, the present study suggests that stroke has a negative impact on the function of the stomatognathic system, especially concerning left temporal muscle activity at rest, tongue pressure, and the distribution of occlusal forces in the first permanent molars.

## Supporting information

S1 File Data from the 12 subjects of the control group. (PDF)

S2 File Data from the 12 subjects of the stroke group. (PDF)
