# Supplementary material for: Stroke: An electromyographic approach to the masseter and temporal muscles, orofacial soft tissue pressure, and occlusal force
Source: PLoS One. 2023 Mar 1;18(3):e0282362. doi: 10.1371/journal.pone.0282362 (PMC9976995; doi:10.1371/journal.pone.0282362)
Supplement: S1 File — (PDF) [file pone.0282362.s001.pdf]

Control	Mandibular							
	Rest				Protrusion			
	RT	LT	RM	LM	RT	LT	RM	LM
1	0,830266	0,824479	0,162725	0,417146	0,704843	0,739866	0,428076	0,514248
2	0,109063	0,373048	0,153272	0,125296	0,606759	0,52651	0,351359	0,728658
3	0,520563	0,565369	1,075463	0,259609	0,356428	0,781939	0,618842	0,213865
4	0,146346	0,221776	0,067077	0,148203	0,130686	0,313445	0,423733	0,624358
5	0,233844	0,576159	0,315443	0,175012	0,679547	0,97351	0,96775	2,936878
6	0,396196	0,364537	0,352017	0,182229	0,186036	0,080793	0,205795	0,406785
7	0,169347	0,186064	0,924341	1,448845	0,23099	0,208914	1,403372	0,661367
8	0,45071	0,119519	0,047949	0,066747	0,204856	0,044612	0,078223	0,08544
9	1,220072	0,920978	0,137159	0,330234	0,261026	1,183116	0,439632	0,440846
0	0,053261	0,032711	0,127179	0,025622	0,053469	0,044746	0,376778	0,51233
11	0,102323	0,413611	0,199381	0,166422	0,054198	0,52651	0,097205	0,189748
12	0,528982	0,365078	0,907637	0,972807	0,431523	0,198004	0,776939	0,621884

Tasks - EMG								
Right Laterality				Left Laterality				
RT	LT	RM	LM	RT	LT	RM	LM	Tongue
0,577724	0,719402	0,33478	0,57603	0,427845	0,541913	0,627902	0,309986	38
0,107911	0,463754	0,155363	1,066982	0,537634	0,029451	0,32208	0,140793	73
0,427195	0,089975	1,013218	0,228012	0,271582	0,24507	0,82384	1,000943	54
0,166853	0,301866	0,05633	0,225361	0,181767	0,411547	0,279128	1,178406	55
0,31046	0,84106	0,845574	0,177296	0,339774	1,006623	0,755767	0,162453	72
0,158084	0,068943	0,201011	0,350081	0,112619	0,13584	0,338117	0,010339	28
0,440608	0,13032	1,141834	1,422442	0,513531	1,427621	0,686618	1,193894	34
0,749237	0,148664	0,171777	0,301019	0,483349	0,617282	0,490527	0,363212	52
0,264176	0,463754	0,30536	0,400289	0,40234	0,815614	0,489623	0,27621	47
0,372099	0,035507	0,093719	0,137309	0,046916	0,048941	0,209377	0,019684	69
0,372099	0,75	0,079421	0,101753	0,110521	0,818966	0,20391	0,05566	72
0,518742	0,18337	1,030788	0,586921	0,484549	0,484036	0,934802	0,509874	74

Pressure			Oclusal Force		
Right Buccinator Musc	Left Buccinator Musc	Lips	Right Side	Left Side	16
18	21	13	61,9	38,1	29,8
21	24	32	61,4	38,6	33,8
29	23	32	60,4	39,6	21,6
21	22	18	78,2	21,8	19,3
26	27	29	42,7	57,3	16
20	19	17	51,1	48,9	4,7
20	23	33	40,8	59,2	27,9
28	29	39	41,7	58,3	16
23	26	21	55,3	44,7	9,1
25	25	19	44,2	55,8	22,4
27	25	47	33,1	66,9	15,75
18	22	21	61,5	38,5	16

Molar Force

26	36	46
13,3	13,3	30,4
11,6	11,6	36,6
15,5	16	14,7
13,6	16,5	17,6
15	12,7	20,7
25,5	16	32,5
24,6	24,6	30,4
15	16	22,9
29,7	29,7	20
27,9	36,3	33,3
28,8	33	17,95
15	16	20
