# Supplementary material for: Stroke: An electromyographic approach to the masseter and temporal muscles, orofacial soft tissue pressure, and occlusal force
Source: PLoS One. 2023 Mar 1;18(3):e0282362. doi: 10.1371/journal.pone.0282362 (PMC9976995; doi:10.1371/journal.pone.0282362)
Supplement: S2 File — (PDF) [file pone.0282362.s002.pdf]

	Mandibular							
Stroke	Rest				Protrusion			
	RT	LT	RM	LM	RT	LT	RM	LM
1	0,984902	0,159398	0,077525	0,396771	0,253108	0,537041	0,542803	0,381776
2	0,105231	0,100179	0,07061	0,127407	0,323662	0,046512	0,151956	0,447636
3	0,952707	0,221898	0,083455	0,334149	0,856066	0,279708	1,292643	0,953029
4	0,130832	0,118549	0,073895	0,065641	0,120114	0,068679	0,223047	0,247093
5	0,389347	0,189061	0,454456	0,112807	1,07743	0,149029	0,597969	0,175679
6	0,061881	0,0426	0,143342	0,073979	0,196306	0,092627	0,154281	0,054233
7	0,277266	0,373263	0,55985	0,219415	0,395098	0,776307	1,063591	0,472041
8	0,358399	0,431656	0,191914	0,413481	0,748871	0,88331	0,986865	0,893575
9	0,299772	0,400554	0,816879	0,533172	0,37576	0,3614	0,683121	0,387761
0	0,130305	0,197763	0,178748	0,090848	0,056302	0,10867	0,237951	0,341267
11	0,289206	0,190093	0,078122	0,515888	0,441955	0,191787	0,254514	0,974766
12	0,099463	0,138915	0,097939	0,148979	0,173792	0,148585	0,453353	0,527639

Tasks - EMG								
Right Laterality				Left Laterality				
RT	LT	RM	LM	RT	LT	RM	LM	Tongue
0,27087	0,112931	0,073864	1,745098	0,585702	0,630356	0,74495	0,522491	68
0,281161	0,074112	0,137762	0,416289	0,15141	0,145285	0,158326	0,418305	28,33
1,028101	0,255766	0,082174	0,305998	0,533242	0,244672	1,063141	0,188648	45,33
0,509983	0,071766	0,052033	0,126705	0,191992	0,079965	0,146564	0,092191	20,66
1,756452	0,151011	0,423001	0,223286	1,001098	0,479192	0,522281	0,190686	8,66
0,209634	0,072201	0,210109	0,017176	0,327494	0,103987	0,308186	0,167433	47,66
0,184169	0,563865	0,493766	0,244678	0,208005	0,46724	0,766833	0,413852	49,66
0,473628	0,568131	0,636472	0,744974	0,474341	0,777896	0,74275	1,641703	26
0,658435	0,403326	1,07431	0,677674	0,339666	0,677755	0,665074	0,87216	38,66
0,145873	0,25849	0,065465	0,625545	0,103434	0,247703	0,179127	0,397251	11
0,366599	0,182684	0,150922	0,761682	0,3111	0,156435	0,069188	1,207477	37
0,320945	0,141038	0,429524	0,464719	0,122449	0,541038	0,242338	0,33181	19

Pressure			Oclusal Force		Molar	
Right Buccinator Musc	Left Buccinator Musc	Lips	Right Side	Left Side	16	26
35	39	43,33	9,9	90,1	0	21,4
18,66	16,33	41,33	53,6	46,4	36	14,7
28,33	26	31,33	43,2	56,8	3,5	14,5
16	22	31,33	41,6	58,4	13,9	17,3
14,33	17,33	17,33	45,6	54,4	5,9	1,4
11	13,66	15,33	8,3	91,7	0	3,8
18,66	22,66	32	59	41	8,1	15
12,33	17	15,33	50	50	0	0
12,66	11,66	15,66	74,8	24,2	15,8	0
7,66	10,33	15,5	9,6	90,4	0	0
22,33	20,33	17,66	48,1	51,9	26,9	5,5
32	33	17,66	31,5	68,5	0,7	2,6

Force	
36	46
21,4	0
23	36
13,8	3,5
17,9	13,7
1,4	10,8
24,2	0
11,3	13,8
12,7	37,5
0	20,6
0	0
6	25,4
3	0,7
